# Elemental Composition of PM_2.5_ and PM_10_ and Health Risks Assessment in the Industrial Districts of Chelyabinsk, South Ural Region, Russia

**DOI:** 10.3390/ijerph182312354

**Published:** 2021-11-24

**Authors:** Tatyana G. Krupnova, Olga V. Rakova, Kirill A. Bondarenko, Artem F. Saifullin, Darya A. Popova, Sanja Potgieter-Vermaak, Ricardo H. M. Godoi

**Affiliations:** 1 Institute of Natural Sciences and Mathematics, South Ural State University, 454080 Chelyabinsk, Russia; rakovaov@susu.ru (O.V.R.); et2142bka04@susu.ru (K.A.B.); et1932saf02@susu.ru (A.F.S.); et1834pda85@susu.ru (D.A.P.); 2 Ecology & Environment Research Centre, Department of Natural Science, Manchester Metropolitan University, Manchester M1 5GD, UK; S.Potgieter@mmu.ac.uk; 3 Molecular Science Institute, University of the Witwatersrand, Johannesburg 2000, South Africa; 4 Environmental Engineering Department, Federal University of Parana, Curitiba 80060-240, Brazil; rhmgodoi@ufpr.br

**Keywords:** PM_10_ and PM_2.5_, trace elements, industry emissions, health risk

## Abstract

Air pollution impacts all populations globally, indiscriminately and has site-specific variation and characteristics. Airborne particulate matter (PM) levels were monitored in a typical industrial Russian city, Chelyabinsk in three destinations, one characterized by high traffic volumes and two by industrial zone emissions. The mass concentration and trace metal content of PM_2.5_ and PM_10_ were obtained from samples collected during four distinct seasons of 2020. The mean 24-h PM_10_ ranged between 6 and 64 μg/m^3^. 24-h PM_2.5_ levels were reported from 5 to 56 μg/m^3^. About half of the 24-h PM_10_ and most of the PM_2.5_ values in Chelyabinsk were higher than the WHO recommendations. The mean PM_2.5_/PM_10_ ratio was measured at 0.85, indicative of anthropogenic input. To evaluate the Al, Fe, As, Cd, Co, Cr, Cu, Mn, Ni, Pb, and Zn concentration in PM_2.5_ and PM_10_, inductively coupled plasma mass spectrometry (ICP-MS) was used. Fe (337–732 ng/m^3^) was the most abundant component in PM_2.5_ and PM_10_ samples while Zn (77–206 ng/m^3^), Mn (10–96 ng/m^3^), and Pb (11–41 ng/m^3^) had the highest concentrations among trace elements. Total non-carcinogenic risks for children were found higher than 1, indicating possible health hazards. This study also presents that the carcinogenic risk for As, Cr, Co, Cd, Ni, and Pb were observed higher than the acceptable limit (1 × 10^−6^).

## 1. Introduction

Globally, an increasing awareness of air quality and air pollutants in general are fostered amongst populations due to media coverage, changing policies, new air quality standards, and disaster events such as the COVID-19 pandemic. As such, urban populations become increasingly aware of the impact that poor air quality has on their health and the environment [1]. Most countries have adopted air quality guidelines and in 2010, the Russian Federation approved the legislation for the maximum permissible concentrations of atmospheric particles with aerodynamic diameter < 2.5 μm (PM_2.5_) and <10 μm (PM_10_) (35 and 60 μg/m^3^ (24-h mean concentrations), 25 and 40 μg/m^3^ (annual mean concentrations), respectively) [2]. This evidently resulted in numerous monitoring stations across the country. It needs to be noted though, that the adopted guideline values are much higher than what has been recommended by the WHO, which recently changed to even lower levels of 15 and 45 μg/m^3^ (24-h mean concentrations), and 5 and 15 μg/m^3^ (annual mean concentrations), for PM_10_ and PM_2.5_, respectively [3].

The focus of this research is on a typical Russian industrial city, where urban air quality is further impacted by local industry. For example, it has been shown that mechanical engineering industries contributes up to 13% of ambient PM_2.5_, ferrous metallurgy up to 79% of PM_2.5_, and non-ferrous metallurgy up to 43% of PM_2.5_ [4]. Concentrations of PM_2.5_ and PM_10_ in the ambient air near industrial enterprises often exceed hygienic standards. This is most concerning as Russia currently reports an increase in industrial production. It is therefore not surprising that residents are worried about smog episodes driven by elevated particulate matter (PM) levels, especially in industrialized urban environments. If one considers the usual urban air pollution sources [5] in addition to industry emissions, it becomes evident that continuous site-specific air quality monitoring, source apportionment, and data analyses are of cardinal importance for residents’ well-being and health.

A potential way of source apportionment is to investigate the ratio of PM_2.5_ to PM_10_ (PM_2.5_/PM_10_) as it can provide information on the origin and production processes [6,7]. Not only does it inform as to the predominant size of the PM, but could be an indication if the PM is predominantly anthropogenic (higher ratio) or naturally occurring airborne particles (lower ratio) [8]. In addition, the PM_2.5_/PM_10_ ratio has been shown to provide useful information on local dusty processes in the atmosphere and types of PM pollution in a particular region [9,10,11,12,13,14]. Apart from mass concentrations, the PM collected from industrial city areas is enriched with trace elements (TEs) [15,16,17]. Due to the difference in chemistry that these particles will exhibit, it is important to analyze the chemical profile of PM on a site-specific basis, so as to predict potential health risks of the inhabitants.

As general continuous air quality monitoring is limited to the last decade in Russia, only a few studies [17] have been performed using one-year continuous PM data. Even fewer studies report the chemical profile and TEs in PM and atmospheric aerosol (Table S1). Mitigation strategies need to be informed by reliable long-term monitoring and analysis. For that reason, the main objectives of the study were (1) to identify the spatiotemporal variations in PM_2.5_, PM_10_, and PM_2.5_/PM_10_ in a typical industrial Russian city, Chelyabinsk; (2) determine the concentration of TEs in PM_2.5_ and PM_10_ collected in the Chelyabinsk urban area; (3) assess non-carcinogenic and carcinogenic health risks associated with the inhalation of PM.

To the authors’ knowledge, this is the first time that the spatiotemporal variation of the metal(loid) concentrations in PM_2.5_ and PM_10_ in a typical Russian industrial city has been investigated systematically (120 samples analyzed during four seasons and at three different stations) for a time period of one year.

## 2. Materials and Methods

### 2.1. Study Area, Data and Samples Collection

**Figure 1:**
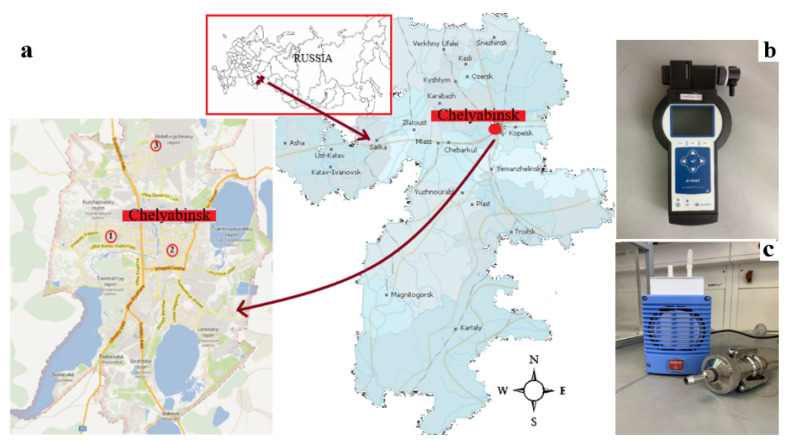
The area under study and the observation instruments used to record data. (**a**) Chelyabinsk (Russia) and the location of the three sampling stations. (**b**) Atmas (NTM Protection, Moscow, Russia) device, providing simultaneous measurement of the hourly data of PM_2.5_ and PM_10_. (**c**). The aerosol cascade impactor sampler.

The three sampling stations were located in urban residential areas in Chelyabinsk (55°09′14″ N, 61°25′44″ E, Elevation: 219 m) (Figure 1). Chelyabinsk is located on the eastern slope of the Southern Urals. The city has a humid continental climate. The average temperature in January is well below freezing point (−14 °C). Mid-summer temperatures are relatively cool (19 °C), while the annual average is a few degrees above freezing point at 3 °C, indicating a moderate climate for Russia. The population of Chelyabinsk during the last census (2010) was 1.130 million. The city has a land area of roughly 530 km^2^.

Chelyabinsk experiences heavy air pollution with about 120 days per year identified as high pollution days. The three most dangerous air pollutants in Chelyabinsk are formaldehyde, HF, and NO_2_ [5].

Station 1 was located near highways with heavy automotive traffic. Station 2 and Station 3 were industrial sites located near metallurgical plants. The Atmas device (NTM Protection, Moscow, Russia) measures PM_2.5_ and PM_10_ mass concentrations based on unipolar corona charging and electrostatic detection [18]. The analyzer consists of a 2.5 and 10 µm cutoff diameter impactors for PM_2.5_ and PM_10_ fractional measurements. Ground-based observations of hourly PM_2.5_ and PM_10_ mass concentrations were obtained from January 2020 to December 2020.

In addition, 72-h PM_2.5_ and PM_10_ samples were collected on polycarbonate filters (Sartorius, Göttingen, Germany) with diameter of 25 mm and a pore size of 0.4 µm using low volume 4 stage cascade impactor samplers (IKS-4, Ekaterinburg, Russia) operated at a flow rate of 16 L· min^−1^ at a height of 2 m. The following size 4 fractions could be collected: >10 µm, 10–5 µm, 5–2.5 µm, and <2.5 µm. The PM_2.5_ and PM_10_ samples were collected at each station for each of the four seasons in 2020 (12–30 January, 6–27 April, 9–25 July, and 5–30 October).

### 2.2. Sample Pretreatment and Chemical Composition Analyses

The loaded PM_2.5_ and PM_10_ filters were placed into a polytetrafluoroethylene (PTFE) digestion vessel for acid treatment (2 mL hydrofluoric acid and 6 mL nitric acid), then microwave digested (MWS 4 Speedwave, Berghof, Germany) for 2 h after the setup routine to analyze metal(loid) elements [19,20,21]. After digestion, the extracts were filtered using a blue ribbon filter (Whatman Grade 589/3 ashless filter paper), and distilled water was added such that the total volume was 25 mL. The major (Al and Fe) and trace elemental compositions (As, Cd, Co, Cr, Cu, Mn, Ni, Pb, and Zn) were analyzed using iCAP 7200 (Thermo Fisher, Waltham, MA, USA) Inductively Coupled Plasma Optical Emission Spectrometry (ICP-OES) and Perkin Elmer ELAN 9000 Inductively Coupled Plasma Mass Spectrometry (ICP-MS), respectively.

For quality control purposes the following certified reference materials were used: GSO 10413-2014 CO cespitose and podsolic srednesuglinisty soil (I BEND Rosselkhozakademiya’s VNIIA, Russia), GSO 7186-95 of loess soil (Bronnitsky geological and geochemical expedition Institute of Mineralogy, Geochemistry and Crystal Chemistry of Rare Elements, Russia), and GSO 3486-86 of aluminosilicate loose deposits (Vinogradov Institute of Geochemistry SB RAS, Russia). For quality assurance/quality control, 10 blank filters and standards were digested and analyzed as described above to serve as method blanks. For each element the limit of detection (LOD) has been estimated considering the standard deviation of 10 blank measurements (three times the standard deviation was used). The detection limits in ng/m^3^ on a 72 h measurement period and the recovery test results are presented in Table 1. The range of recovery efficiency was 87–119%. Reproducibility was tested by analyzing the same standard solution 10 times.

### 2.3. Exposure Assessment and Risk Calculation

The health impact from exposure to the elements in PM_2.5_ and PM_10_ is used in the US Environmental Protection Agency (USEPA) human health evaluation method [22]. There are three major exposure pathways to metal(loid)s: ingestion, inhalation, and dermal contact [19,23,24]. In the present study, we assessed the health risk from inhalation [25] according to USEPA standard [22,26]. The non-carcinogenic risk of nine elements As, Cd, Co, Cr, Cu, Mn, Ni, Pb, and Zn was estimated.

The average daily dose (*ADDinh*) (mg kg^−1^ day^−1^) for elements via inhalation was calculated as [27]:

(1) $$ADD_{inh}=C\cdot \frac{InhR\cdot EF\cdot ED}{BW\cdot AT},$$

where the *ADDinh* is the average daily dose (mg kg^−1^ day^−1^) of exposure to elements through inhalation; *C* is the concentration of elements, (mg m^−3^); *InhR* is the inhalation rate of PM_2.5_; *EF* is exposure frequency; *ED* is exposure duration; *BW* is average body weight; *AT* is average time with *AT_non-car_*. A detailed description of the values of exposure factors for children and adults applied to the Equation (1) is given in Table 2.

As some of the components differ between cities (countries) it is necessary to alter those values [32,33]. Unfortunately, we did not find information about body weight, for example.

The non-carcinogenic health risk of exposure to elements from PM_2.5_ in Chelyabinsk is determined as the hazard quotient (*HQ*inh) [29,30,31]:

(2) $$HQinh=\frac{ADDinh}{RfD}.$$

In this equation, *RfD* is an estimation of maximum permissible risks to the human population through daily exposure with consideration of sensitive groups (children) during their lifetime.

To assess the cumulative potential non-carcinogenic effects posed by many contaminants, the total exposure hazard index, which is the summation of all the individual hazard quotient, was calculated the *HQ*inh value of each target chemical was summed:

(3) HI=HQinh1+HQinh2+⋯+HQinhn

If *HQ*inh or *HI* < 1, a population is located in a safe area, whereas potential non-carcinogenic effects would occur in case *HQ*inh or *HI* > 1.

The *CRA* (carcinogenic risk assessment) for individual elements was calculated [29,30,31,32]:

(4) $$LADD_{inh}=C\cdot \frac{InhR\cdot EF\cdot ED}{BW\cdot AT},$$

(5) CRA=LADDinh·CSFinh,

where *AT* is average time with *AT_car_* and *CSFinh* is the slope factor.

Carcinogenic risk is the probability of an individual developing any form of cancer from lifetime exposure to carcinogenic hazards. The recommended level of *CRA* < 1 × 10^−6^ can be regarded as negligible, whereas *CRA* > 1 × 10^−4^ is likely to lead to health issues. The acceptable or tolerable risk for regulatory purposes ranges from 1 × 10^−6^ to 1 × 10^−4^ [29,30,31,32,33,34,35,36].

Arsenic, Cd, Co, Ni, and Pb were treated as potential carcinogenic contaminants, whereas the other elements were regarded as non-carcinogenic according to the classification groups defined by USEPA [26]. Chrome Cr(VI) is more toxic than Cr(III) and only Cr(VI) is considered as a carcinogen. Therefore, the *CSFinh* and *RfD* of Cr(VI) were assumed as for total Cr to assess the worst situation of Cr [37].

### 2.4. Scanning Electron Microscopy

SEM analysis was performed on a Jeol JSM-7001F Scanning Electron Microscopy Complex, 30 keV, EDS Oxford INCA X-max 80, WDS Oxford INCA WAVE, EBSD and HKL (JEOL Ltd., Tokyo, Japan), equipped with standard automated features such as autofocus/stigmator, auto gun, and auto contrast with multiple live image display. SEM-EDS is a non-destructive analytical method for surface elemental analysis, with a potential detection limit of 0.1–0.5 wt.% for most elements [38,39,40,41]. A thin layer of gold was deposited on the surface of each sample in order to achieve better conductivity and less electron charge.

## 3. Results and Discussion

### 3.1. Pollution Characteristics of Atmospheric Particulate Matter

**Figure 2:**
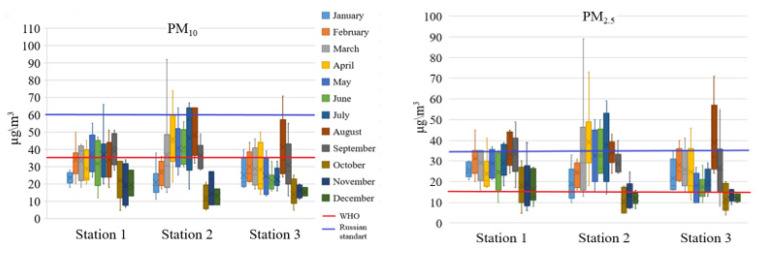
24-h PM_10_ and PM_2.5_ concentration measured in 2020 by the Atmas device.

Figure 2 shows the 24-h means of recorded PM_2.5_ and PM_10_ levels for each month of 2020.

The mean PM_10_ ranged between 6 and 64 μg/m^3^. PM_2.5_ levels were reported from 5 to 56 μg/m^3^. About 3% of PM_10_ and 30% of PM_2.5_ values exceeded the Russian standard values (35 and 60 μg/m^3^, respectively) by 1.1 to 1.7 times. It should be noted that Russian standards differ from WHO recommended limits. The WHO guidelines state that annual average concentrations of PM_2.5_ and PM_10_ should not exceed 5 and 15 µg/m^3^, while 24-h average exposures should not exceed 15 and 45 µg/m^3^, respectively [3]. About half of the 24-h PM_10_ and most of the PM_2.5_ values in Chelyabinsk were higher than the WHO recommendations.

An increase of PM concentration during the spring-summer period is not surprising as it is generally the dry season how it can be seen in Figure S1a. What is interesting though, is that despite the increase in precipitation during August, the levels of PM_10_ and PM_2.5_ at Station 3 were quite high, pointing towards additional site sources during this time. Long-range transport from nearby forest fires may also have contributed to the mass concentrations during this period. More than 600 forest fires were registered in close vicinity of the Chelyabinsk region in the summer of 2020 [42]. Figure S2 shows that there is a significant difference in both the magnitude of the area in which fires were reported as well as the number of fires when compared to 2019. Usually, forest fires in the Urals begin in April and end in October, peaking in May (Figure S2, 2019). However, in 2020, forest fires in the Sverdlovsk region (located north of Chelyabinsk) raged throughout the summer, with a maximum in July. The prevailing wind directions in summer 2020 were north and northwest (Figure S2c). It gave possibility of the smoke plume fromforest fires in the Sverdlovsk region to influence on the PM concentrations. In addition, summer inversions occur often, and between 8 and 12 days are typical night-time inversion occurrences, all of which may contribute to increased PM mass concentrations [43,44]. There is the possibility of similar inversions during autumn and winter months, albeit less frequent, which could potentially also play a role in higher sporadic concentrations [43,44]. During the summer of 2020 there were 28 days with low wind speeds (less than 1 m per second) [45], which would further exacerbate pollution.

At all the stations, the PM_2.5_ and PM_10_ levels decreased substantially during the autumn-winter period starting from October. Chelyabinsk is one of the snowiest cities in the Russian Federation. The decrease of PM concentration in winter can partially be explained by the “wash-out” effect, as the first snowfall typically happens in October and are lasting throughout the winter [46,47,48]. Snow grains to moderate snow has been reported from January–March and October–December of 2020 (Figure S1b). The established snow cover prevents dusting of urban soils, as well as resuspension of dust from roads and sidewalks (see pictures of the city in winter in Figure S1). The variation in PM mass concentration levels at Station 1, situated furthest from industrial activities, could most probably be ascribed to changes in traffic flow and natural occurrences. It should be noted that traffic volume varied only slightly because this is a transport site in a residential area. On the other hand, the level of PM mass concentration at Stations 2 and 3, which is overall higher than at Station 1, is probably due to the industrial emission influence. Station 2 was located near a large slag dump of a metallurgical enterprise. Station 3 was located within the zone of industrial emissions of a metallurgical plant. The substantial increase in PM_2.5_ and PM_10_ levels from March to August at Station 2, compared to Stations 1 and 3, could be due to dust resuspension of the nearby slag dump caused by windy dry weather. During the autumn-winter period rains [46] and snow cover [47,48] significantly reduced dust episodes and the levels at Station 2 is comparable to that of Stations 1 and 3. The PM_2.5_ and PM_10_ concentrations observed during the spring-summer period of 2020 at Station 3 were substantially lower than at Station 2 and markedly lower than at Station 1. These lower levels correlate with the COVID-19 lockdown period during that time. On the other hand, the significant increase in both PM_2.5_ and PM_10_ during August correlated with an increase in plant production, after lockdown was lifted.

**Figure 3:**
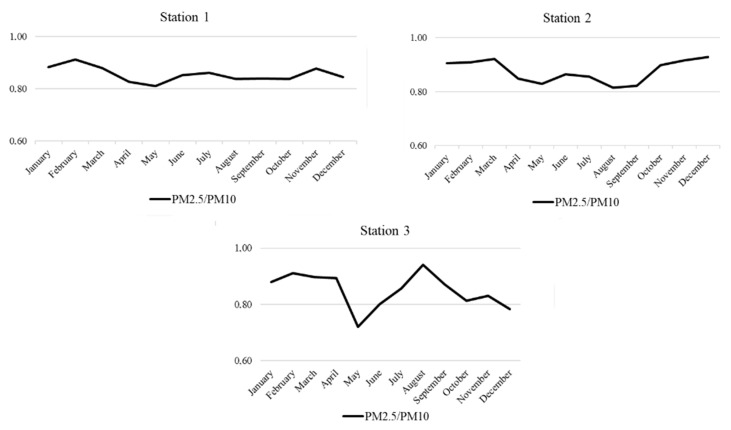
PM_2.5_/PM_10_ ratio levels.

Figure 3 shows that the average PM_2.5_/PM_10_ ratio was 0.85, and the minimum and maximum ratio 0.70 and 0.95, respectively.

The average PM_2.5_/PM_10_ ratio obtained in Chelyabinsk was generally higher than reported for Asia (0.5) [9,14,49], China (0.62) [10,11,12], and in 20 European Cities (0.6) ([50] and references therein). Seasonal variation (often with a diurnal distribution) is also observed and differs according to meteorological conditions. For example, in Wuhan the ratio decreases from spring through summer and increases again from autumn through winter, while in Africa the opposite is observed. The data in Figure 3 shows a near constant ratio at Station 1, and a more prominent seasonal distribution for Station 2. This is indicative of constant sources of PM throughout the year, where differences in seasonal meteorological conditions could explain the fluctuations in ratios. On the other hand, the ratio at Station 3 did not stay constant and did not display the typical seasonal variation. This could be due to the lockdown period during which it is reasonable to assume that the PM_2.5_ levels would drop significantly as a result of a drop in vehicle emissions and the nearby steel production plant. It is also evident from Figure 2 that the PM_2.5_ levels during the lockdown months were substantially lower. This conclusion is further supported by the significant rise in the ratio during August–September when production at the plant increased. According to Statistics Department data [51], in May 2020 metallurgical production of the Chelyabinsk region decreased to 77.6% of the previous year’s production. After the slump in production during the May-June-July period, a gradual increase in August and September was observed, although still lower than the previous year. In fact, for September, the level of production was 90.2% and in November it exceeded the previous year’s production by 1.8%.

Table 3 presents the results of elemental composition of PM_2.5_ and PM_10_. The results reveal that Al (192–324 ng/m^3^) and Fe (337–732 ng/m^3^) concentrations constitute the major components. Both Al and Fe are normally assigned to crustal origin (natural sources) but could also be partially from anthropogenic origin, for example road dust resuspension (likely at all stations), slag dump dust (Station 2), and steel manufacturing plant emissions (Station 3 with nearly double the Fe concentration in the PM_10_ fraction). Zn (77–206 ng/m^3^), Mn (10–96 ng/m^3^), and Pb (11–41 ng/m^3^) had the highest concentration among the TEs, all of which could also be identified as potentially toxic elements and of anthropogenic origin [19]. There were no statistically significant season differences observed or a discernable difference between the two fractions, therefore the often observed enrichment of TEs in the smaller fraction was not evident in our data set. The Cd, As, Co, and Cr concentrations were quite low and near the detection limits at all monitoring stations.

Table 4 shows the concentrations found in Chelyabinsk compared to the data reported in other cities around the world. In general, the levels of the metals investigated in Asia are significantly higher than in our study. In fact, the data from our study compares surprisingly well with Brazil and European cities, apart from Zn which is substantially higher. The latter may be an indication of successful application of emission treatment processes, scrubbing the particulate matter released.

### 3.2. SEM-EDS Analysis

**Figure 4:**
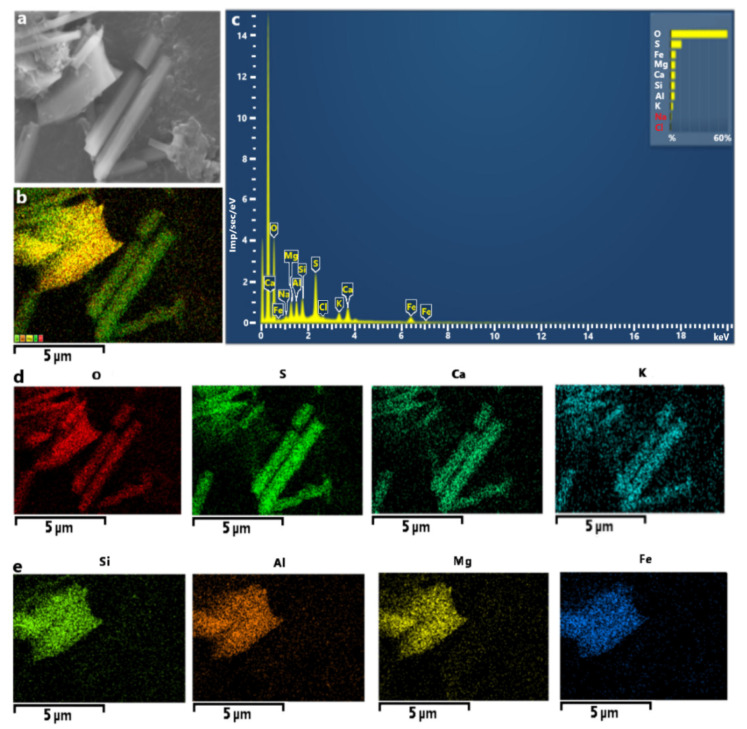
(**a**) SEM micrograph (magnification 500×), (**b**) chemical mapping of the most abundant elements, and (**c**) EDS spectrum of aggregate particles from the aluminosilicate group (**d**), maps rod-like particles consisting of K, Ca, S, and O that could be potassium and calcium sulfates (**e**), and plates consisting of Fe-Mg-enriched aluminosilicates.

As the highest concentrations of TEs were observed in PM collected at Station 1, further investigation was performed, using SEM-EDS. Information about the shape and composition of single particles can provide some insight in potential pollution sources as well as the fate of these particles upon inhalation [41]. According to the characteristic SEM-EDS analysis, atmospheric particles collected in the winter of 2020 from the Chelyabinsk urban area (Station 1) contained 20 elements (Al, B, C, Ca, Cl, Cr, Cu, Fe, K, Mg, Mn, N, Na, Ni, O, Pb, S, Si, Ti, and Zn) with concentrations more than the detection limit (0.1 wt.%). The most abundant elements were Fe and Mg which were present in 80–100% of the particles. These particles could be natural occurring aluminosilicates, which is indeed indicated in Figure 4e. This is not surprising as Chelyabinsk stands on sedimentary rocks and granite, typical for the Urals, consisting of oxides Al_2_O_3_ (14–15%), SiO_2_ (70–72%), Fe_2_O_3_ (0.7–1.1%), and MgO (0.6–1.1%) [61].

**Figure 5:**
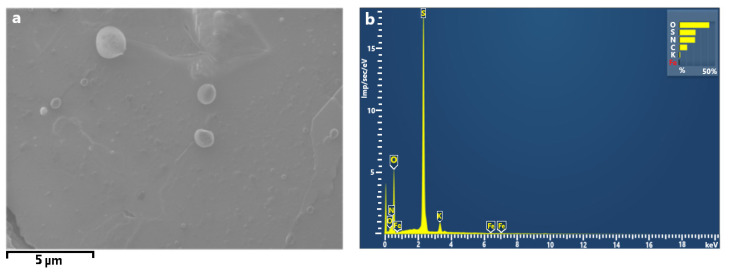
C-rich particles, (**a**) SEM micrograph (magnification 500×) and (**b**) EDX spectrum.

Figure 5 shows the predominant morphologies of carbon-rich (C-rich) particles collected at Station 1. They were regular spherical and spheroidal shapes, with some presenting surface defects such as porosity. The particle size of this type varied in the range of 1–5 μm. These particles are observed in cities with high vehicular traffic density and could be associated with exhaust emissions from automobiles using gasoline or diesel combustibles, as well as fly ash originating from coal-fired power stations [62].

**Figure 6:**
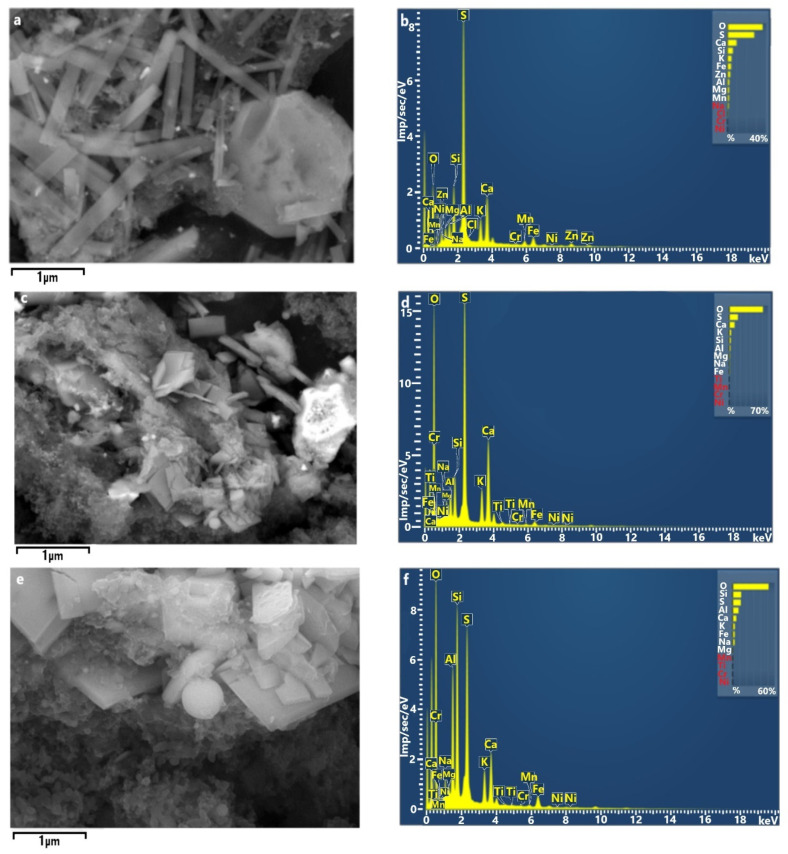
SEM micrograph S-rich rod-shaped, crystalline, and spherical (magnification 5000×, 3000×, and 10,000×) particles (**a**,**c**,**e**, respectively) and their EDS spectrum (**b**,**d**,**f**, respectively).

Station 1 had the largest number of sulfur-rich (S-rich) particles, with more than half of them containing more than 3% sulfur. Sulfate PM_2.5_ is commonly identified as markers of secondary aerosols related to long-distance transport [63,64,65,66,67]. The majority of the sulfate particles had one or more potentially toxic metal inclusions. Figure 6 shows typical rod-shaped, crystalline, and spherical particles. The size of metal-containing S-rich particles was about 1 µm. Most metal particles were classified as Fe-rich (e.g., hematite), Zn-rich (e.g., zinc sulfate and zinc oxide), Pb-rich (e.g., anglesite), Mn-, or Pb-rich, which were likely emitted from road traffic (exhaust and tire, brake, car body, or road surface abrasions) [68]. Station 1 had the highest traffic volume and one can conclude that source of the metals is mostly traffic-related. Metals such as Fe and Zn can be linked to the corrosion of car-body parts. Zn, and Pb can be mostly related to brake-pad erosion; Fe, Cu, Pb, and Zn from brake-disc wear. Road dust and roadside soil often contain metals, including Pb, Cu, Cd, and Zn, indicative of contamination by road traffic emissions and the abrasion of road surfaces. It has been shown that sulfates from aqueous SO_2_ (S(IV)) oxidation catalyzed by transition metals are an important atmospheric process during winter, an alternative to the photochemical pathway that is highly unlikely because of the ultralow O_3_ concentrations [68]. Metal catalysis can promote the conversion of SO_2_ to sulfates in fog droplets [69]. The internal mixing of metals and acidic constituents solubilize metals and modify metal inclusion shapes. The solubilization of metals in airborne particles can increase their toxicity in the particles [70].

**Figure 7:**
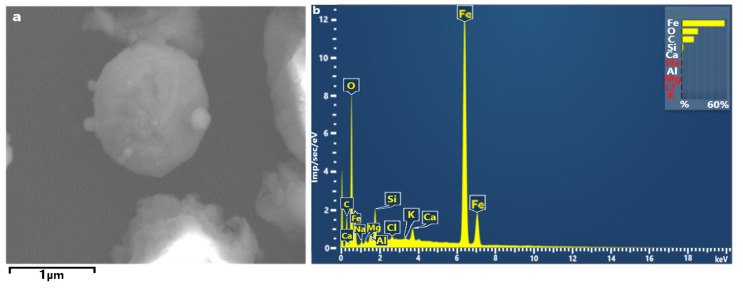
(**a**) SEM micrograph Fe-rich particles (magnification 6000×) and (**b**) their EDS spectrum.

Figure 7 shows spherical Fe-rich PM_2.5_ found at Stations 2 and 3. These particles consisted of iron oxide. They could be steel furnace dust emitted by the metallurgical enterprises which is dumped in numerous slag waste dumps in the Chelyabinsk urban area near Stations 2 and 3. Their size ranges between 0.5 and 2 μm and they present a peculiar morphology; they are characterized by perfect sphericity indicating their smelting iron origin or metallurgical activities in general.

### 3.3. Health Risk Assessment of Airborne Metal(loid)s

Exposure to PM_2.5_ bound metal(loid)s may pose serious carcinogenic or non-carcinogenic toxicity in humans depending on various factors such as exposure concentration, duration, and frequency. Health risk assessments of TEs through the inhalation pathway for both children and adult were determined, and the values of *HQinh* and *HI* are reported in Table 5.

It was observed that the non-carcinogenic risk for each individual TE was well below the safe level (*HQinh* = 1) for both groups (adults and children), except *HQinh* (Mn) value for children at Stations 2 and 3. It was higher than 1 for stations located near metallurgical plants. Exposure to all other TEs through inhalation was within safe limits for all stations. The *HQinh* of all studied TEs indicated a greater hazard to children than adults. Table 5 shows that HI values were lower than 1 for adults but risks for children were significant. The greatest danger was posed by metallurgical industrial PM emissions containing Mn.

Arsenic, Cr, Cd, Co, Ni, and Pb were considered as carcinogens. The *CRA* was determined for children and adults and reported in Table 6. The *CRA* values for Cr and As had higher carcinogenic potential in both populations. The carcinogenic risk of each TEs for both groups were in the range of <1 × 10^−6^. The total *CRA* of all TEs was slightly higher for adults than for children. At Station 1 it was 1.06 × 10^−5^ and 1.53 × 10^−5^ for children and adults, respectively. It shows the high carcinogenic potential in both populations.

## 4. Conclusions

In this study the PM_2.5_, PM_10_, the PM_2.5_/PM_10_ ratio, and the concentrations of major elements (Al and Fe) and TEs (As, Cd, Co, Cr, Cu, Mn, Ni, Pb, and Zn) in PM_2.5_ and PM_10_ were determined in an industrialized city, Chelyabinsk, Russia for the period January 2020–December 2020. The new WHO guideline values for 24-h PM mass concentrations were exceeded during all seasons for PM2.5, while the PM10 mass concentrations exceeded it about half the time. The PM levels were lowest in winter. Mass concentrations at Stations 2 and 3 are substantially higher than at Station 1 and could be ascribed to the nearby industrial emissions. The average fine particle ratio (PM_2.5_/PM_10_) was 0.8 on average for the recording period, indicating anthropogenic origin. The concentrations of major components in PM were Al (192–324 ng/m^3^) and Fe (337–732 ng/m^3^) while Zn (77–206 ng/m^3^), Mn (10–96 ng/m^3^), and Pb (11–41 ng/m^3^) had the highest concentrations among TEs.

SEM-EDS allowed us to provide information about the sources of PM and their potential toxic characteristics. Fe-rich metal particles were observed in industrial polluted stations. Fly ash and S-rich particles were ubiquitous at Station 1, characterized by heavy traffic flows. There is evidence of acid S-rich particles which seem to have solubilized metals from resuspended road dust.

PM_2.5_-producing industrial processes and transport may contribute to excess exposure of the two population groups (adults and children) to metals. Total non-carcinogenic risk values for children were more than 1 and on average 2.7 times higher than non-carcinogenic risk for adults. The common carcinogenic risk upon inhalation (considering As, Cr, Co, Cd, Ni, and Pb) were higher than 10^−6^ but less than 10^−4^ for children and adults. It was indicative of a medium cancer risk during their lifetime. The risk values for adults were slightly higher.

This study shows the importance of site-specific long-term monitoring in dynamic urban environments, such as industrialized cities. The contribution to PM levels form specific sources can be identified, facilitating mitigation steps. In addition, the human health risk can be determined. The latter can inform health professionals and residents, which will empower them to make informed lifestyle choices and diagnosis. The results suggest that measures to reduce air pollution should be implemented through the effective and efficient implementation of urban environment management planning to maintain acceptable urban air quality. The observed concentrations and profiles provide new insights into the sources and dispersion of different types of particle pollution illustrating the importance of adopting sustainable air quality strategies in urban planning of Russian cities.

## Figures and Tables

**Table 1:** Limits of detection and recovery rate for different elements.

	LOD (ng/m^3^)	Recovery (%)
Al	0.1	97
As	0.3	98
Cd	0.5	109
Co	0.1	98
Cr	1.1	87
Cu	1.8	110
Fe	1.1	105
Mn	0.4	95
Ni	1.4	89
Pb	4.2	119
Zn	0.1	107

LOD is limit of detection.

**Table 2:** Values of exposure factors for elemental doses for children and adults.

Factor	Unit	Value		References
		Children	Adults	
*EF*	days/year	350	350	[26,28,29]
*ED*	years	6	24	[22,26,28,29]
*BW*	kg	15	70	[22,26,28,29,30]
*AT_non-carc_*	days	365 × ED	365 × ED	[22,28,29,30]
*AT_carc_*	days	365 × 70	365 × 70	[26,28,29]
*InhR*	m^3^/days	7.6	12.8	[22,26,28,29,31]

EF is exposure frequency; ED is exposure duration; BW is average body weight; *AT_non-car_* and *AT_carc_* are average times for non-carcinogens and for carcinogens, respectively; *InhR* is the inhalation rate.

**Table 3:** The annual average concentrations (ng/m^3^) of metal(loid)s in PM_2.5_ and PM_10_ at recording stations in Chelyabinsk.

	PM_2.5_			PM_10_		
Metal(loid)	Station 1 (*n* = 20)	Station 2 (*n* = 19)	Station 3 (*n* = 21)	Station 1 (*n* = 20)	Station 2 (*n* = 19)	Station 3 (*n* = 21)
Al	192 ± 42	303 ± 66	268 ± 58	324 ± 71	319 ± 66	324 ± 71
As	9.2 ± 3.6	2.2 ± 0.9	1.4 ± 0.6	9.2 ± 3.9	6.1 ± 2.5	1.9 ± 0.8
Cd	1.0 ± 0.4	0.7 ± 0.3	0.6 ± 0.2	1.0 ± 0.5	0.6 ± 0.3	0.7 ± 0.3
Co	0.2 ± 0.1	0.4 ± 0.1	0.4 ± 0.1	0.2 ± 0.1	0.1 ± 0.1	0.4 ± 0.1
Cr	2.5 ± 0.4	3.5 ± 0.6	3.0 ± 0.5	8.4 ±1.3	1.7 ± 0.3	3.6 ± 0.6
Cu	11 ± 2	6.5 ± 1.1	5.7 ± 1.0	13 ±2	5.4 ± 0.8	8.0 ± 1
Fe	417 ± 98	693 ± 163	651 ± 153	337 ± 79	474 ± 101	732 ± 173
Mn	21 ± 5	35 ± 9	30 ± 8	96 ± 24	10 ± 3	38 ± 10
Ni	3.4 ±1.0	1.5 ± 0.4	1.4 ± 0.4	3.2 ± 1.0	1.7 ± 0.5	1.6 ± 0.5
Pb	27 ± 5	15 ± 3	11 ± 2	41 ± 9	24 ± 5	15 ± 3
Zn	115 ± 19	147 ± 25	142 ± 21	206 ± 34	77 ± 12	144 ± 24

**Table 4:** Comparison between metal(loid)s (ng/m^3^) and PM_2.5_ (μg/m^3^) concentrations at different sites in the world.

Area	PM_2.5_ (24-h Mean)	Al	As	Cd	Co	Cr	Cu	Fe	Mn	Ni	Pb	Zn	Refs
Standard Limits	15		6	5						20	50		[3,52]
Agra (India)	131–189	1388–1688	17–35	22–26	3–4	309–354	190–210	3440–4290	58–82	61–67	320–670	319–758	[53]
Nanjing (China)	281	1662 *	28 *	8.4 *	1.6 *	42 *	137 *	1163 *	123 *	27 *	448 *	878 *	[54]
Kanpur (India)	172	109	16	34	-	52	627	308	114	7	318	408	[55]
Chelyabinsk (Russia)	5–56	192–303	1.4–9.2	0.6–1.0	0.2–0.4	1.5–3.5	5.7–11	417–493	21–35	1.4–3.4	11–27	115–147	This study
Curitiba (Brazil)	9.2 *					1.7 *	2.2*				8.05 *	4.3 *	[56]
Manaus (Brazil)	9.2 *					3.1 *	9.6*				12.1 *	19.9 *	[56]
Frankfurt (Germany)			0.4–1.8		0.1–0.8	4.4–17	13–121		4.6–40	2.3–10	0.6–46		[57]
Salentum Peninsula (Italy)	6–92	1.7–207				0.1–13.5	0.1–31.3	0.9–416.9	0.1–8.4	0.2–30.4	0.9–65.7	2.1–154.1	[58]
Paris (France)		33 *		0.16 *		3.6 *	36 *	128 *	3.1 *	1.5 *	5.6 *	28 *	[59]
Athens (Greece)				1 *		11 *	41 *	1024 *	19 *	11 *	16*		[60]

* Mean values.

**Table 5:** The values of hazard quotient (*HQinh*) for children and adults in various stations.

TE	Children			Adults		
	Station 1	Station 2	Station 3	Station 1	Station 2	Station 3
As	1.49 × 10^−2^	3.55×10^−3^	2.26 × 10^−3^	5.35 × 10^−3^	1.28 × 10^−3^	0.82 × 10^−3^
Cd	4.86 × 10^−2^	3.40 × 10^−2^	2.92 × 10^−2^	1.75 × 10^−2^	1.23 × 10^−2^	1.05 × 10^−2^
Co	1.70 × 10^−2^	3.40 × 10^−2^	3.40 × 10^−2^	6.14 × 10^−3^	1.23 × 10^−2^	1.23 × 10^−2^
Cr	4.25 × 10^−2^	5.95 × 10^−2^	5.09 × 10^−2^	1.53 × 10^−2^	2.15 × 10^−2^	1.84 × 10^−2^
Cu	1.33 × 10^−4^	7.86 × 10^−5^	6.89 × 10^−5^	4.80 × 10^−5^	2.84 × 10^−5^	2.49 × 10^−5^
Mn	0.71	1.19	1.02	0.26	0.43	0.37
Ni	1.18 × 10^−1^	5.21 × 10^−2^	4.86 × 10^−2^	4.26 × 10^−2^	1.88 × 10^−2^	1.75 × 10^−2^
Pb	6.56 × 10^−2^	3.64 × 10^−2^	2.67 × 10^−2^	2.37 × 10^−2^	1.32 × 10^−2^	0.97 × 10^−2^
Zn	0.93 × 10^−3^	1.19 × 10^−3^	1.15 × 10^−3^	0.34 × 10^−3^	0.43 × 10^−3^	0.42 × 10^−3^
Hazard Index (*HI*)	1.02	1.41	1.21	0.37	0.51	0.44

**Table 6:** The calculated values of carcinogenic risk for adults and children in the present study.

Metals	Children			Adults		
	Station 1	Station 2	Station 3	Station 1	Station 2	Station 3
As	5.75 × 10^−6^	1.37 × 10^−6^	8.74 × 10^−7^	8.29 × 10^−6^	1.98 × 10^−6^	1.26 × 10^−6^
Cd	2.62 × 10^−7^	1.84 × 10^−7^	1.57 × 10^−7^	3.79 × 10^−7^	2.65 × 10^−7^	2.27 × 10^−7^
Co	8.16 × 10^−8^	1.63 × 10^−7^	1.63 × 10^−7^	1.18 × 10^−7^	2.36 × 10^−7^	2.36 × 10^−7^
Cr (VI)	4.27 × 10^−6^	5.98 × 10^−6^	5.12 × 10^−6^	6.16 × 10^−6^	8.63 × 10^−6^	7.39 × 10^−6^
Ni	1.68 × 10^−7^	7.43 × 10^−8^	6.94 × 10^−8^	2.43 × 10^−7^	1.07 × 10^−7^	1.00 × 10^−7^
Pb	4.72 × 10^−8^	2.62 × 10^−8^	1.92 × 10^−8^	6.82 × 10^−8^	3.79 × 10^−8^	2.78 × 10^−8^
Total risk	1.06 × 10^−5^	7.80 × 10^−6^	6.41 × 10^−6^	1.53 × 10^−5^	1.13 × 10^−5^	9.25 × 10^−6^

## Data Availability

Data sharing is not applicable to this article.

## Supplementary Materials

The following are available online at https://www.mdpi.com/article/10.3390/ijerph182312354/s1, Figure S1: Effect of snowfalls and snow cover on the decrease of air pollution in Chelyabinsk during winter comparing with summer 2020: relative humidity (a) and precipitation (b) in Chelyabinsk during 2020 according [11], Figure S2: Effect of forest fires on the air pollution in Chelyabinsk during summer 2020: number (a) and area of forest fires (b) on the Sverdlovsk region in 2020 compared with 2019 according to [12], wind rose in Chelyabinsk during June-August 2020 (c) according to [11], the map of studied area (d), Table S1: Summary of conducted studies in Russian Federation on metal(loid)s contamination of atmospheric aerosol.
