# Supplementary material for: Elemental Composition of PM2.5 and PM10 and Health Risks Assessment in the Industrial Districts of Chelyabinsk, South Ural Region, Russia
Source: Int J Environ Res Public Health. 2021 Nov 24;18(23):12354. doi: 10.3390/ijerph182312354 (PMC8657131; doi:10.3390/ijerph182312354)
Supplement: Supplementary file 1 [file ijerph-18-12354-s001.zip › ijerph-1429284-supplementary.pdf]

Supplementary material

# Elemental Composition of $PM_{2.5}$ and $PM_{10}$ and Health Risks Assessment in the Industrial Districts of Chelyabinsk, South Ural Region, Russia

**Tatyana G. Krupnova [1,*], Olga V. Rakova [1], Kirill A. Bondarenko [1], Artem F. Saifullin [1], Darya A. Popova [1], Sanja Potgieter-Vermaak [2,3] and Ricardo H. M. Godoi [4]**

[1] Institute of Natural Sciences and Mathematics, South Ural State University, 454080 Chelyabinsk, Russia; rakovaov@susu.ru (O.V.R.); et2142bka04@susu.ru (K.A.B.); et1932saf02@susu.ru (A.F.S.); et1834pda85@susu.ru (D.A.P.)
[2] Ecology & Environment Research Centre, Department of Natural Science, Manchester Metropolitan
University, Manchester M1 5GD, UK; S.Potgieter@mmu.ac.uk
[3] Molecular Science Institute, University of the Witwatersrand, Johannesburg 2000, South Africa
[4] Environmental Engineering Department, Federal University of Parana, Curitiba 80060-240, Brazil;
rhmgodoi@ufpr.br
* Correspondence: krupnovatg@susu.ru; Tel.: +7-964-2444419

Table S1. Summary of conducted studies in Russian Federation on metal(loid)s contamination of atmospheric aerosol

| Studied region, time | Type of studied particles | Metal(loid)s | Pollution source(s) | Reference |
|---|---|---|---|---|
| Moscow, one observation station in the center of Moscow (Pyzhevsky per., 3), from March 25 to May 3, 2020 | Total aerosol, $PM_{10}$ and $PM_{2.5}$ concentrations | 1) concentration metal(loid)s in aerosol every day<br>2) Concentration metal(loid)s in <0.5, 0.5–1.5, 1.5–2.5, 2.5–4.0, 4.0–6.5, and >6.5 µm cascade – average for all time of study | The sampling site was located in the administrative center of the city in the vicinity of motorways with medium and light traffic loads, but away from industrial and power enterprises. In late March 2020, the **anomalously high** mass concentrations of different particle fractions and all elements accumulated in aerosols were caused by **unfavorable meteorological conditions**; the **anticyclone dominating** over the Moscow region brought air masses from neighboring regions with biomass **fires**. | [1] |
| Peterhof observation station on the southwestern border of St. Petersburg, from January 2013 to December 2017 | Organic and elemental carbon total aerosol concentrations | Not studied | The sampling site was located in a sparsely populated area (~80 000 people). M**ostly biogenic sources were identified, b**ut there were **individual periods** when it was showed the **anthropogenic origin** of the recorded episodes of aerosol growth were caused by the intense accumulation of air pollution during **unfavorable meteorological conditions (calm anticyclone weather).** | [2] |
| Meteorological Observatory of Moscow State University spring period of 2017 and 2018 | Particulate black carbon (BC) concentration | Not studied | The pollution sources were emissions from diesel fuel in motor vehicle **transport** | [3] |
| Meteorological Observatory of Moscow | $PM_{10}$ and BC concentration | Not studied | The pollution sources were emissions from diesel fuel in motor vehicle **transport** | [4] |

| | | | | |
|---|---|---|---|---|
| State University, from 1 April to 31 May 2018. | | | | |
| Moscow, three observation stations: in the center of Moscow (Pyzhevsky per., 3), Meteorological Observatory of Moscow State University, and in the semi-background suburban zone, June 10 - July 10, 2019 | Total aerosol and $PM_{2.5}$ concentration | Concentration metal(loid)s in <0.5, 0.5–1.5, 1.5–2.5, 2.5–4.0, 4.0–6.5, and >6.5 µm cascade – average for all time of study | The results of elemental analysis of aerosols have confirmed that **road transport** is currently the main pollution source in the Moscow region | [5] |
| Russian Arctic, from 1985 to the present time | Total aerosol concentration and BC concentration | Concentration metal(loid)s in PM – seemingly TSP [7, 8] | The Arctic has few local sources of aerosol emissions; however, the physicochemical composition of the Arctic atmosphere is impacted strongly by outflows of different pollutants from Eurasian and North American midlatitudes. It means the long-range transports of submicron aerosol of **natural and anthropogenic origins**, the lifetime of which reaches a week or longer. | [6-8] |
| Tsimlaynsk raion (Rostov oblast), August 2–15, 2012 | Total aerosol concentration | Concentration metal(loid)s in <0.5, 0.5–1.5, 1.5–2.5, 2.5–4.0, 4.0–6.5, and >6.5 µm cascade - two samples were collected during the observation period: between August 2 and 9 and between August 9 and 15, 2012. | It was shown that the soils of Kalmykia (mainly the **sandy soil**) can serve as the source of aerosol. There was an increase in the content of aerosol in the atmosphere due to the transfer of salt and dust arid aerosol from Kalmykia and addition of aerosol from the industrial areas of Ukraine. | [10] |

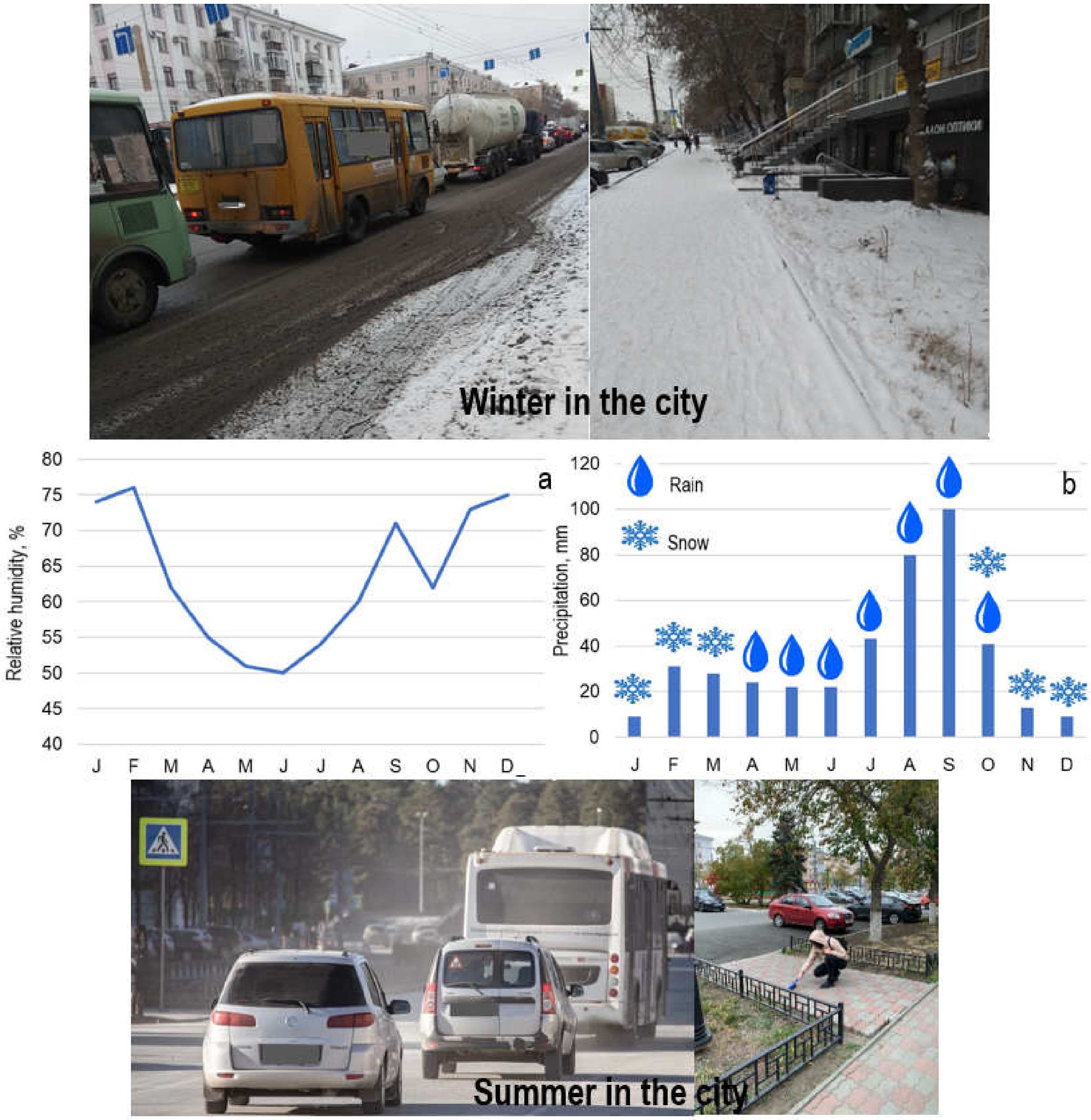


Figure S1. Effect of snowfalls and snow cover on the decrease of air pollution in Chelyabinsk during winter comparing with summer 2020:
relative humidity (a) and precipitation (b) in Chelyabinsk during 2020 according [11]

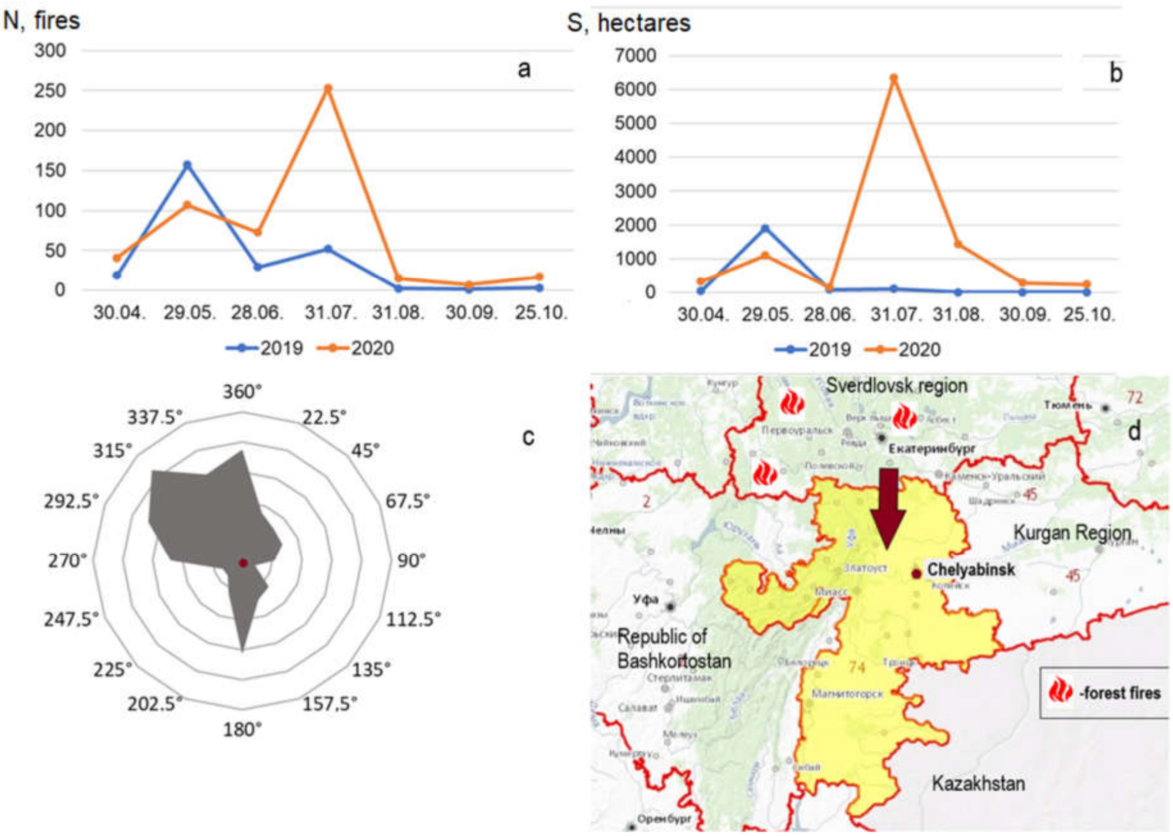


Figure S2. Effect of forest fires on the air pollution in Chelyabinsk during summer 2020: number (a) and area of forest fires (b) on the Sverdlovsk region in 2020 compared with 2019 according to [12], wind rose in Chelyabinsk during June-August 2020 (c) according to [11], the map of studied area (d)

**References**


1. Gubanova, D.P.; Vinogradova, A.A.; Iordanskii, M.A., Skorokhod, A.I. Time Variations in the Composition of Atmospheric Aerosol in Moscow in Spring 2020. *Izvestiya - Atmospheric and Ocean Physics* **2021**, *57(3)*, 297-309. DOI: 0.1134/S0001433821030051
2. Vlasenko, S.S.; Volkova, K.A.; Ionov, D.V.; Ryshkevich, T.I.; Ivanova, O.A.; Mikhailov, E.F. Variation of Carboneceous Atmospheric Aerosol Near St. Petersburg. *Izvestiya - Atmospheric and Ocean Physics* **2019**, *55(6)*, 619-627. DOI: 10.1134/S0001433819060161
3. Popovicheva, O.B.; Volpert, E.; Sitnikov, N.M.; Chichaeva, M.A.; Padoan, S. Black carbon in spring aerosols of Moscow urban background. *Geography, Environment, Sustainability* **2020**, *13(1)*, 233-243. DOI: 10.24057/2071-9388-2019-90
4. Chubarova, N.E.; Androsova, E.E.; Kirsanov, A.A.; Vogel, B.; Vogel, H.; Popovicheva, O.B.; Rivin, G.S. Aerosol and its radiative effects during the aeroradcity 2018 Moscow experiment. *Geography, Environment, Sustainability* **2019,** *12(4)*, 114-131. DOI: 10.24057/2071-9388-2019-72
5. Gubanova, D.P.; Elansky, N.F.; Skorokhod, A.I.; Kuderina, T.M.; Iordansky, M.A.; Sadovskaya, N.V.; Anikin, P.P. Physical and chemical properties of atmospheric aerosols in Moscow and its suburb for climate assessments. *IOP Conference Series: Earth and Environmental Science* **2020,** *606(1)*, art. no. 012019. DOI: 10.1088/1755-1315/606/1/012019

6. Shevchenko, V.P.; Starodymova, D.P.; Vinogradova, A.A.; Lisitzin, A.P.,; Makarov, V.I.; Popova, S.A.; Sivonen, V.V.; Sivonen, V.P. Elemental and organic carbon in atmospheric aerosols over the northwestern coast of Kandalaksha Bay of the White Sea. *Doklady Earth Sciences* **2015,** *461(1)*, 242-246. DOI: 10.1134/S1028334X1503006X
7. Vinogradova, A.A.; Malkov, I.P.; Polissar, A.V.; Khramov, N.N. Elemental composition of the surface atmospheric aerosol in the Arctic regions of Russia. *Izvestiya - Atmospheric & Oceanic Physics* **1993**, *29(2)*, 149-157.
8. Shevchenko, V.; Lisitzin, A.; Vinogradova, A.; Stein, R. Heavy metals in aerosols over the seas of the Russian Arctic. *Science of the Total Environment* **2003,** *306(1-3)*, 11-25. DOI: 10.1016/S0048-9697(02)00481-3
9. Sakerin, S.M.; Kabanov, D.M.; Makarov, V.I.; Pol'kin, V.V.; Popova, S.A.; Chankina, O.V.; Pochufarov, A.O.; Radionov, V.F.; Rize, D.D. Spatial Distribution of Atmospheric Aerosol Physicochemical Characteristics in the Russian Sector of the Arctic Ocean. *Atmosphere* **2020**, *11*, 1170. https://doi.org/10.3390/atmos11111170
10. Artamonova, M.S.; Gubanova, D.P.; Iordanskii, M.A.; Lebedev, V.A.; Maksimenkov, L.O.; Minashkin, V.M.; Obvintsev, Y.I.; Chketiani, O.G. Variations of the aerosol concentration and chemical composition over the arid steppe zone of Southern Russia in summer. *Izvestiya - Atmospheric and Ocean Physics* **2016,** *52(8)*, 769-783. DOI: 10.1134/S000143381608003X
11. Chelyabinsk weather archive. Available online: https://rp5.ru (accessed on 01 November 2021)
12. Daily summary of forest fires. Available online: https://mprso.midural.ru/news/show/id/810 (accessed on 01 November 2021)
